# Supplementary material for: Persistence of Mating Suppression of the Indian Meal Moth Plodia Interpunctella in the Presence and Absence of Commercial Mating Disruption Dispensers
Source: Insects. 2020 Oct 14;11(10):701. doi: 10.3390/insects11100701 (PMC7602279; doi:10.3390/insects11100701)
Supplement: Supplementary file 1 [file insects-11-00701-s001.pdf]

Table S1. Effect of entrainment, photophase, and light conditions on mating in a 25 ml arena.

Entrainment at Time of Pairing	Light Condition at Time of Pairing	Trials	Mated Immediately
Photophase	Light	6	6
	Dark	6	4
	Fisher's Exact Test: $p = 0.454$		
Scotophase	Light	6	6
	Dark	6	6
	Fisher's Exact Test: $p = 1.0$		

Table S2. Effect of photophase and light conditions on mating status of females in a 950 ml arena.

Part of Photoperiod at Time of Pairing	Light Condition at Time of Pairing	Trials	Spermatophore Found
Photophase	Light	8	7
Scotophase	Dark	7	7

Not significant, Fisher's Exact Test, $p = 1$.

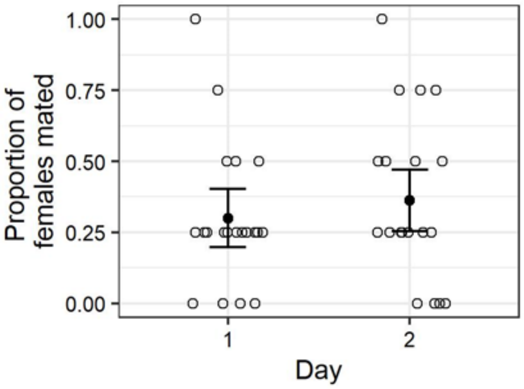

Figure S1. The mean number of females mated was slightly higher on day 2 of experiment 2 (36% mated) vs day 1 (30%) mated. However, the number of barrels with 0, 1, 2, 3, 4, or 5 mated females did not differ significantly between the two days of the experiment (Fisher's Exact test, P = 0.877).
